# Implementation and experimental validation of a robust hybrid direct aperture optimization approach for mixed‐beam radiotherapy

**DOI:** 10.1002/mp.15258

**Published:** 2021-10-14

**Authors:** Emily Heath, Silvan Mueller, Gian Guyer, Alisha Duetschler, Olgun Elicin, Daniel Aebersold, Michael K. Fix, Peter Manser

**Affiliations:** ^1^ Carleton Laboratory for Radiotherapy Physics Carleton University Ottawa Canada; ^2^ Division of Medical Radiation Physics and Department of Radiation Oncology Inselspital, Bern University Hospital and University of Bern Bern Switzerland; ^3^ Department of Physics ETH Zurich Zurich Switzerland; ^4^ Center for Proton Therapy Paul Scherrer Institute Villigen Switzerland

**Keywords:** mixed‐beam radiation therapy, modulated electron radiation therapy, robust optimization, treatment planning

## Abstract

**Purpose:**

The objectives of the work presented in this paper were to (1) implement a robust‐optimization method for deliverable mixed‐beam radiotherapy (MBRT) plans within a previously developed MBRT planning framework; (2) perform an experimental validation of the delivery of robust‐optimized MBRT plans; and (3) compare PTV‐based and robust‐optimized MBRT plans in terms of target dose robustness and organs at risk (OAR) sparing for clinical head and neck and brain patient cases.

**Methods:**

A robust‐optimization method, which accounts for translational setup errors, was implemented within a previously developed treatment planning framework for MBRT. The framework uses a hybrid direct aperture optimization method combining column generation and simulated annealing. A robust plan was developed and then delivered to an anthropomorphic head phantom using the Developer Mode of a TrueBeam linac. Planar dose distributions were measured and compared to the planned dose. Robust‐optimized and PTV‐based plans were developed for three clinical patient cases consisting of two head and neck cases and one brain case. The plans were compared in terms of the robustness to 5 mm shifts of the target volume dose as well as in terms of OAR sparing.

**Results:**

Using a gamma criterion of 3%/2 mm and a dose threshold of 10%, the agreement between film measurements and dose calculations was better than 97.7% for the total plan and better than 95.5% for the electron component of the plan. For the two head and neck patient cases, the average clinical target volume (CTV) dose homogeneity index (V95%–V107%) over all the considered setup error scenarios was on average 19% lower for the PTV‐based plans and it had a larger standard deviation. The robust‐optimized plans achieved, on average, a 20% reduction in the OAR doses compared to the PTV‐based plans. For the brain patient case, the CTV dose homogeneity index was similar for the two plans, while the OAR doses were 22% lower, on average, for the robust‐optimized plan. No clear trend in terms of electron contributions was found across the three patient cases, although robust‐optimized plans tended toward higher electron beam energies.

**Conclusions:**

A framework for robust optimization of deliverable MBRT plans has been developed and validated. PTV‐based MBRT were found to not be robust to setup errors, while the dose delivered by the robust‐optimized plans were clinically acceptable for all considered error scenarios and had better OAR sparing. This study shows that the robust optimization is a promising alternative to conventional PTV margins for MBRT.

## 1 INTRODUCTION

Mixed‐beam radiotherapy (MBRT) refers to the combination of intensity‐modulated photon beams with intensity‐ and energy‐modulated electron beams. By exploiting the advantageous dosimetric properties of both modalities, specifically the steep lateral penumbra of photon beams and the rapid distal falloff of electron beams, MBRT plans can achieve a comparable target coverage to photon‐only intensity modulated radiation therapy or volumetric modulated arc therapy (VMAT) while reducing the integral dose and dose to organs at risk (OARs).^1^, ^2^, ^3^, ^4^, ^5^, ^6^, ^7^, ^8^

One of the challenges to the delivery of MBRT plans is the need to collimate the electron beams close to the patient surface to minimize in‐air beam scattering. The delivery of multiple electron apertures requires a more efficient collimation system than conventional electron cutouts. Many of the earlier MBRT studies relied on a custom electron multi‐leaf collimator (MLC),^9^, ^10^ however, more recently the feasibility of using the photon MLC with a shortened source‐to‐surface distance (SSD) has been shown.^11^ The optimization of MBRT plans is also challenging due to the increased degrees of freedom resulting from the availability of multiple beam modalities and energies. Earlier MBRT planning approaches reduced these degrees of freedom for the plan optimization by pre‐selecting the electron beam energies based on the target depth.^4^

Two recent publications^7^, ^8^ addressed the limitations mentioned above by developing direct aperture optimization (DAO) methods that simultaneously consider all the available photon and electron beams and energies. Both studies used the photon MLC to collimate the electron beams at a shortened SSD. Mueller et al.^6^ also verified the deliverability of MBRT plans combining non‐coplanar photon arcs with step and shoot electron apertures using the Developer mode of a TrueBeam linac (Varian Medical Systems, Palo Alto, CA). The gamma passing rate for planar film measurements in an anthropomorphic head phantom was better than 99.2%, using a 2%/2 mm criteria.

Recently, Renaud et al.^12^ investigated the robustness of MBRT plans using conventional PTV margins to translational setup errors. They demonstrated that for certain error scenarios either the clinical target volume (CTV) dose coverage was compromised or that hot spots could occur resulting in a clinically unacceptable dose distribution. Using a previously developed column generation method for MBRT optimization,^8^ they implemented robust‐optimization approaches based on both the stochastic and minimax methods.^13^ These approaches account for uncertainties in the optimization variables by minimizing either the expectation value or the maximum (worst case) value of the objective function, evaluated over all considered error scenarios. When evaluated under the same setup error scenarios as the PTV‐based plans, the robust‐optimized plans were able to maintain the CTV coverage and homogeneity. They also noted that the robust‐optimized plans had better OAR sparing compared to the PTV‐based plans. A comparison of the electron and photon contributions of the PTV‐based and robust‐optimized plans revealed an increase in the electron dose contribution for robust‐optimized plans. A preference for higher electron energies was also noted in the robust‐optimized plans.

A limitation of the above‐mentioned study is that they used a beamlet‐based optimization, which does not account for the influence of the MLC. Therefore, the optimized dose may not accurately predict the delivered dose and delivered plan quality may be degraded. The accuracy of the plan delivery was not verified in this study. Furthermore, the authors compared robust‐optimized and PTV‐based plans for chest wall and soft tissue sarcoma cases only. Previous comparison of MBRT and VMAT^7^ plans have demonstrated the suitability of MBRT for other sites, such as head and neck.

Our group recently developed a hybrid DAO approach^14^ for MBRT which uses both column‐generation and simulated annealing to optimize deliverable plans collimated using the photon MLC. The motivation to combine column generation with simulated annealing is to exploit the advantages of both approaches in order to obtain a faster convergence of the objective function value with number of apertures as well as to obtain a better plan quality. Implementing simulated annealing in the hybrid DAO process allows the possibility to further optimize aperture shapes after they have been determined by the column generation step. On the other hand, the column generation is more computationally efficient and does not require the apertures for each field to be predefined. A comparison of the performance of this hybrid DAO algorithm against column generation alone is the subject of a forthcoming paper.

The objectives of the work presented in this paper were as follows: (1) implement a robust‐optimization method for deliverable MBRT plans using this hybrid DAO framework; (2) perform an experimental validation of the delivery of robust‐optimized MBRT plans; and (3) compare PTV‐based and robust‐optimized MBRT plans in terms of target dose robustness and OAR sparing for clinical head and neck and brain cases.

## 2 MATERIALS AND METHODS

### 2.1 Robust‐optimization framework

**FIGURE 1:**
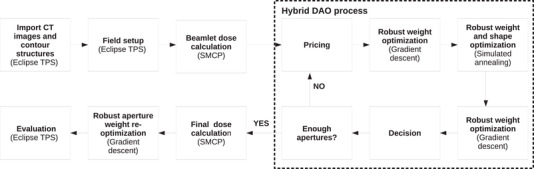
Robust mixed‐beam radiotherapy (MBRT) planning process. The details of each step are described in the text below. The steps within the hybrid direct aperture optimization (DAO) process are repeated until a user‐specified number of apertures have been added

The robust‐optimization method was implemented within a previously developed treatment planning process for MBRT.^14^ Figure 1 shows an overview of the components of the treatment planning process which are described in the following paragraphs. The output of this process is a set of apertures, and their associated monitor units (MUs), defining a step‐and‐shoot MBRT plan.

The robust MBRT planning process begins with importing the CT images and contours into a research version of the Eclipse treatment planning system (version 15.6, Varian Medical Systems, Palo Alto, CA). This is followed by defining the electron and photon fields. It is important to note that for each electron beam direction there are multiple energies available (6, 9, 12, 15, 18, and 22 MeV). Hereafter, an electron “field” refers to a unique beam direction and energy combination.

In the next step, a beamlet dose calculation is performed for each field using the research Eclipse interfaced Swiss Monte Carlo Plan^15^ (SMCP) system. The beamlet dose calculation uses pre‐calculated phase space files which are divided into beamlet‐specific phase space files using a 5 × 5 mm^2^ grid size defined at the isocenter plane. For photon beams, these phase space files are obtained from a VMC++^16^ simulation of the linac head with the MLC and jaws fully retracted. For electron beams, the phase space files are obtained using a multiple source model^17^ which accounts for the jaws and all upstream components. VMC++ is used for the photon beamlet dose calculation in the patient geometry while for the electron beams the macro Monte Carlo algorithm^18^, ^19^, ^20^ is used. To model the dosimetric impact of different setup error scenarios, in the parameters for the beamlet dose calculation the user can specify both systematic and random translational shifts along the three principal axes. Only systematic shifts were investigated in this work and were modeled by applying the same isocenter shift for each incident particle. The output of the beamlet dose calculation is a complete set of beamlets for each user‐specified error scenario plus the “nominal” (no error) scenario.

These beamlet dose distributions are then used as inputs to a hybrid DAO process which uses both column generation and simulated annealing. The optimizer treats the photon and electron apertures equally and the final selection of which modality and energy to add is based on dosimetric characteristics. Starting from an empty aperture pool, the following process is repeated until a user‐defined number of apertures have been generated. At the beginning of each iteration, a set of promising apertures are selected from among all the defined fields based on their “price,” which is determined by the gradient of the objective function with respect to aperture weight^21^ (pricing). In this work, we used a scheme where the most promising aperture is selected for each photon and electron field. Each of the promising apertures is separately added to a copy of the current aperture pool and the following steps are then performed on each aperture pool. After the addition of a new aperture to a given pool, a deterministic weight optimization is performed followed by the removal of any apertures with zero weight. A further refinement of the aperture weights and shapes is then performed using simulated annealing. This is followed by a second deterministic weight optimization. Afterward, the aperture pool that has the lowest objective function value is selected and the other pools are deleted (decision). If the total number of apertures in the remaining pool is equal to the user defined number of apertures, then the hybrid DAO process is terminated. Otherwise, another iteration of the process, starting with the pricing step, is started. Throughout the hybrid DAO process, to enforce robustness against setup errors the pricing, simulated annealing and deterministic weight optimization steps were all modified to use the expectation value of the objective function calculated for the nominal and all user‐specified setup error scenarios.

Following the hybrid DAO process, the dose distributions from the final aperture pool are re‐calculated using SMCP, this time including the influence of the jaws and MLC. For the electron beams, the same multiple source model is used to model the jaws and all upstream components and an in‐house Monte Carlo algorithm, PIN,^22^ is used to model the transport through the MLC. For the photon beams, VMC++ is used to model the transport through the MLC and jaws. The transport in the patient geometry is modeled using the same algorithms as for the beamlet dose calculations. Similar to the beamlet dose calculation, isocenter shifts corresponding to the modeled setup error scenarios were simulated and a complete set of aperture dose distributions are calculated for each user‐specified setup error scenario plus the nominal scenario. These aperture dose distributions are used as inputs to a final robust deterministic weight optimization, whose purpose is to correct for plan deterioration due to discrepancies between the beamlet and final dose calculations. The aperture dose distributions corresponding to each scenario are then summed. The mean statistical uncertainty for the total dose distribution is nominally 0.5%. The dose distributions are then normalized according to the user specification and imported into eclipse for plan evaluation.

### 2.2 Experimental validation

An artificial brain tumor case was created on the CT image of an anthropomorphic head phantom (Alderson Radiation Therapy Phantom, Radiology Support Devices). The CTV contours of a clinical brain case were manually copied while the relevant OARs were delineated by an experienced radiation oncologist. The prescribed dose was 60 Gy in 30 fractions.

**FIGURE 2:**
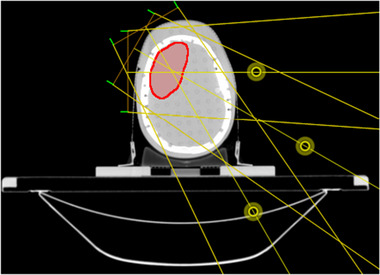
Electron beam arrangements for the artificial brain case on the Alderson head phantom. The location of each beam isocenter is indicated by yellow circle. The clinical target volume (CTV) is shown in red

Three electron beam directions, with SSDs of 67.9 cm, 72.3 cm, and 82.3 cm, were used as shown in Figure 2. The electron beam SSD was aimed to be as close to 70 cm as possible to minimize the in‐air scattering while avoiding collision between the gantry and the patient or couch. Six electron beams were defined for each of the three beam directions, corresponding to energies of 6, 9, 12, 15, 18, and 22 MeV. In addition, seven isocentric 6 MV photon beams were defined. The SMCP beamlet and aperture dose calculations were set to calculate dose to water to be compatible with the dose measurements.

A robust plan comprised of 60 apertures was optimized on the head phantom case considering a systematic 5 mm translational setup error along the superior‐inferior (SI), anterior‐posterior (AP), and left‐right (LR) directions. The equal importance was applied to the nominal and each error scenario when calculating the expectation value of the objective function during the plan optimization. The target volume was the CTV and the plan was normalized so that the *D*_50%_ of the CTV was equal to 60 Gy. Target volume and OAR doses were evaluated on the nominal and six error scenarios and compared to institutional planning guidelines. The plan was deemed acceptable if dose‐volume tolerances were satisfied for all seven scenarios.

Delivery of the robust plan was carried out using the Developer mode of a Varian TrueBeam linac (Varian Medical Systems, Palo Alto, CA). The head phantom was secured to the linac couch with a thermoplastic mask. The phantom setup was verified by acquiring two orthogonal kV images and performing an automated 2D–3D alignment to the planning CT based on bony anatomy. Only translational setup errors were corrected, but it was verified that the rotational errors were less than 1°. Gafchromic EBT3 film (Ashland Advanced Materials, Bridgewater, NJ) was placed between the first and second, and the second and third slices of the head phantom. The film was cut to conform to the outer contour of the phantom. Multiple film irradiations were performed for the delivery of the total plan (photon and electron apertures), photon apertures only and electron apertures only. Each irradiation was performed with and without a 5 mm couch shift (i.e., nominal and one error scenario). Additionally, the dose from the total plan was measured with and without a 5 mm couch shift in the lateral direction in another measurement slice. Shifts along the inferior and lateral directions were selected based on the observed changes in the calculated dose distributions on the measurement slices for these scenarios.

Films were scanned 24 h post‐irradiation using an Epson 10000XL scanner and the FilmQA Pro software (Ashland Advanced Materials, Bridgewater, NJ) following a triple channel dosimetry protocol.^23^ Corrections for lateral scanning artefacts^24^ were applied. The film images and corresponding 2D dose distributions exported from Eclipse were manually aligned using the two phantom support rod holes. The film and dose distribution agreement was assessed using gamma analysis with a 3%/2 mm criterion and a 10% dose threshold, following the recommendations of AAPM Task Group 218.^25^

### 2.3 Planning study

**FIGURE 3:**
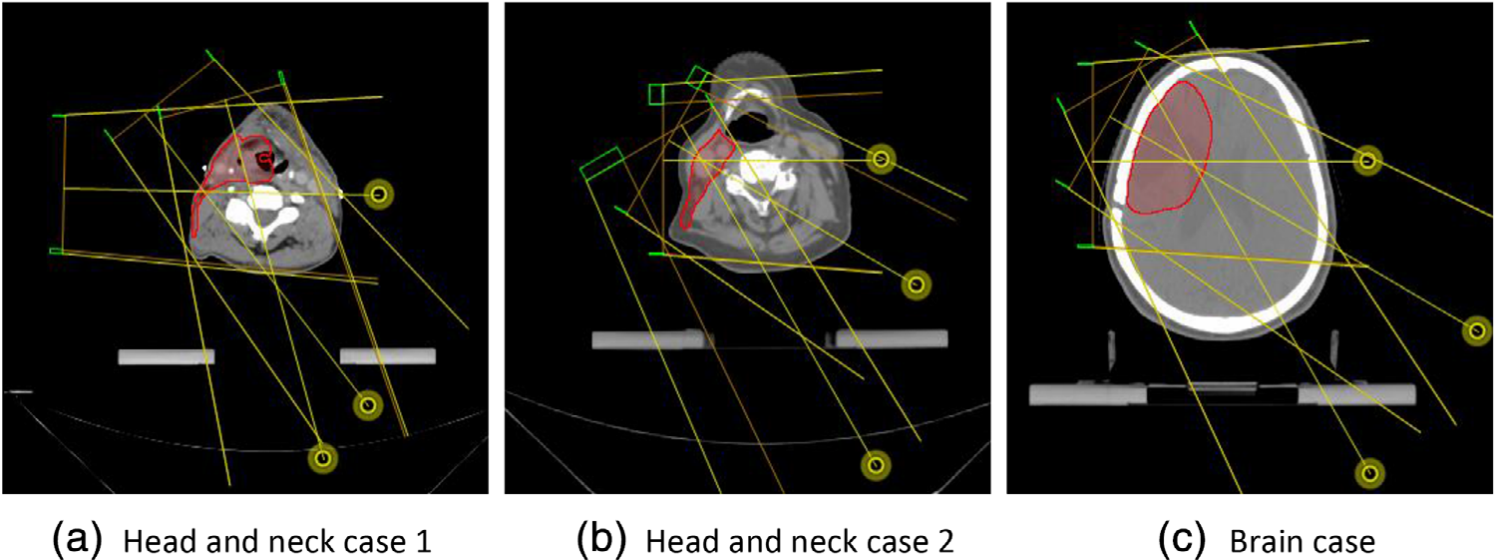
Electron beam arrangements for three clinical cases. The location of each beam isocenter is indicated by yellow circle. The clinical target volume (CTV) for each case is shown in red

Robust‐optimized and PTV‐based MBRT plans were designed on three clinical cases consisting of two head and neck cases (dose prescription: *D*_95%_ = 50 Gy in 25 fractions) and one brain case (dose prescription: *D*_50%_ = 60 Gy in 30 fractions). For the robust‐optimized plans, the dose prescription was applied to the CTV, while for the PTV‐based plans it was applied to the PTV. Both the robust and PTV‐based plans used identical beam arrangements. The electron beam directions specific to each case are shown in Figure 3. These beam directions are selected based on the proximity of the target volume to the patient surface as well as providing an approximately normal incidence of the electron beam on the patient's surface. To avoid introducing any bias due to photon beam angle selection, an identical photon beam arrangement of nine equally angular spaced 6 MV isocentric photon beams were used for all three cases. Dose to medium calculation was used for both beamlet and final aperture dose calculations.

The PTV‐based plans used a 5 mm isotropic CTV to PTV margin. The PTV was cropped 3 mm from the outer body contour following institutional guidelines. For any OARs that had planning at risk volumes (PRVs) specified in the original clinical plan, a 5 mm margin was also used to generate the PRV structure. A normal tissue structure was constructed of all tissue exclusive of the PTV and OARs. Plan acceptability was evaluated according to institutional planning guidelines on the nominal scenario only. The target volume coverage was evaluated on the PTV, while for OARs that had PRVs, the doses to those PRV structures were compared to clinical tolerances.

The robust‐optimized plans were generated using the CTV as the target volume and no PRV structures. For these plans, the normal tissue structure was constructed of all tissue exclusive of the CTV and OARs. Plans were optimized considering systematic 5 mm setup errors in the SI, AP, and LR directions. The equal importance was applied to the nominal and each error scenario when calculating the expectation value of the objective function. Plan acceptability was determined by comparing CTV and OAR doses to institutional planning guidelines on both the nominal and all six error scenarios. The homogeneity of the dose in the CTV was assessed using the percentage of the CTV receiving greater than 95% and less than 107% of the prescribed dose: HI_95/107_ = *V*95% − *V*107%.^26^

The plan quality for both the PTV‐based and robust‐optimized plans was found to depend on the number of apertures. Therefore, for each patient case, the plans were run with up to 200 apertures to determine the required number of apertures for convergence. The resulting PTV‐based and robust‐optimized plans were then compared in terms of target volume coverage and OAR sparing on all seven scenarios.

## 3 RESULTS

### 3.1 Experimental validation

**FIGURE 4:**
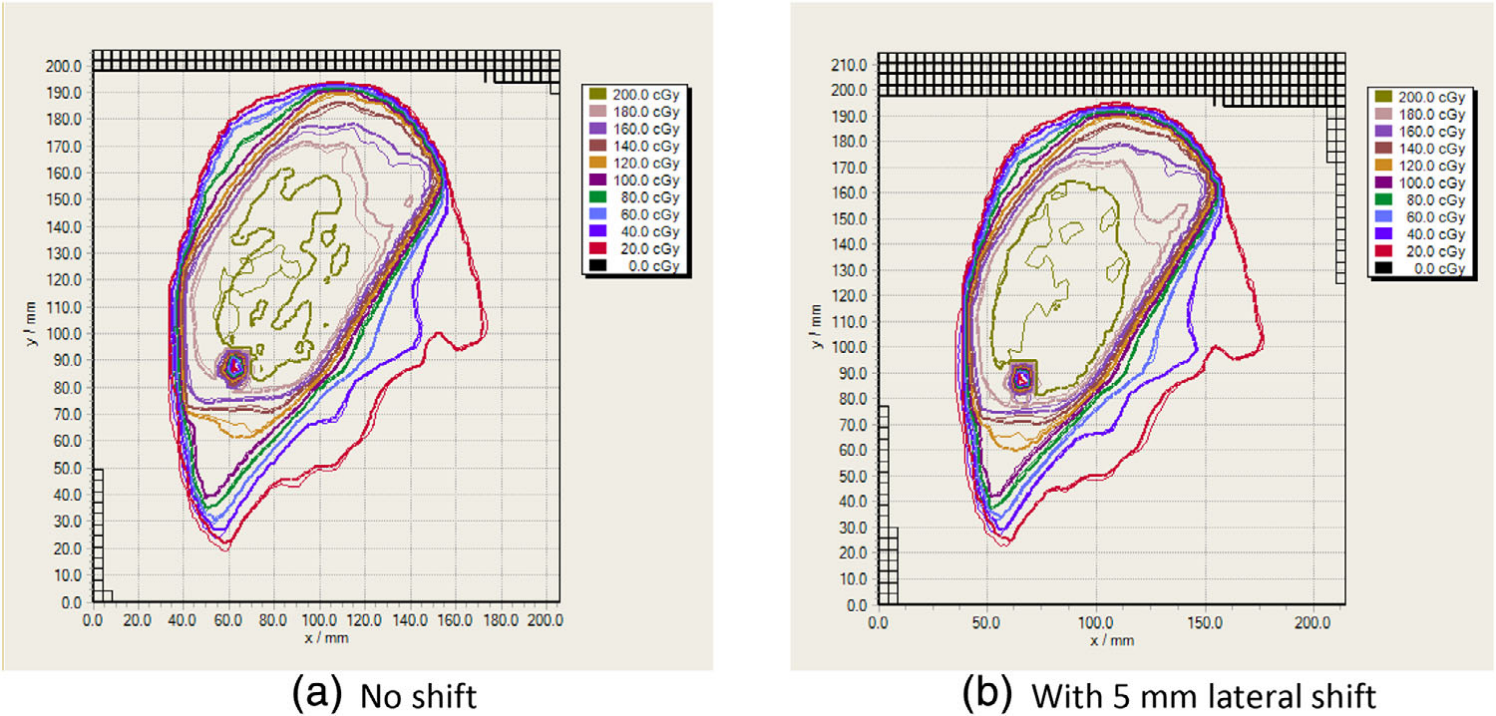
Comparison of measured (thin lines) and calculated (thick lines) isodose distributions for the total plan dose of the robust plan in measurement slice 2. Note that the “hole” located in the lower left quadrant of the isodose distribution corresponds to the location of the Alderson phantom supporting rod and therefore no dose is measured at this location

The results of the gamma comparison of the dose measurements and calculations in the head phantom are shown in Table 1. Delivery of the total plan required 6 min 23 s. A total of eight film measurements were compared with the corresponding dose calculations. When comparing the total plan dose, the agreement was 97.7% or better. The worst agreement was for the electron apertures (95.5%), while the agreement for the photon apertures was 98.7% or better. Figure 4 shows a comparison of measured and calculated isodoses for the same measurement slice, with and without a 5 mm lateral isocenter shift.

**TABLE 1:** Results of gamma comparison (3%/2 mm, 10% threshold) of film measurements and dose calculation for robust plan delivery to head phantom

		**Gamma passing rate (3**%**/2 mm, 10**% **threshold)**		
**Measurement slice (from top of head)**	**Dose Components**	**No couch shift**	**With 5 mm couch shift**	
1	Total	98.1%	Inferior	98.7%
1	Photon	98.7%	Inferior	98.8%
1	Electron	98.0%	Inferior	95.5%
2	Total	97.7%	Lateral	97.7%

### 3.2 Planning study

#### 3.2.1 Head and neck case 1

**FIGURE 5:**
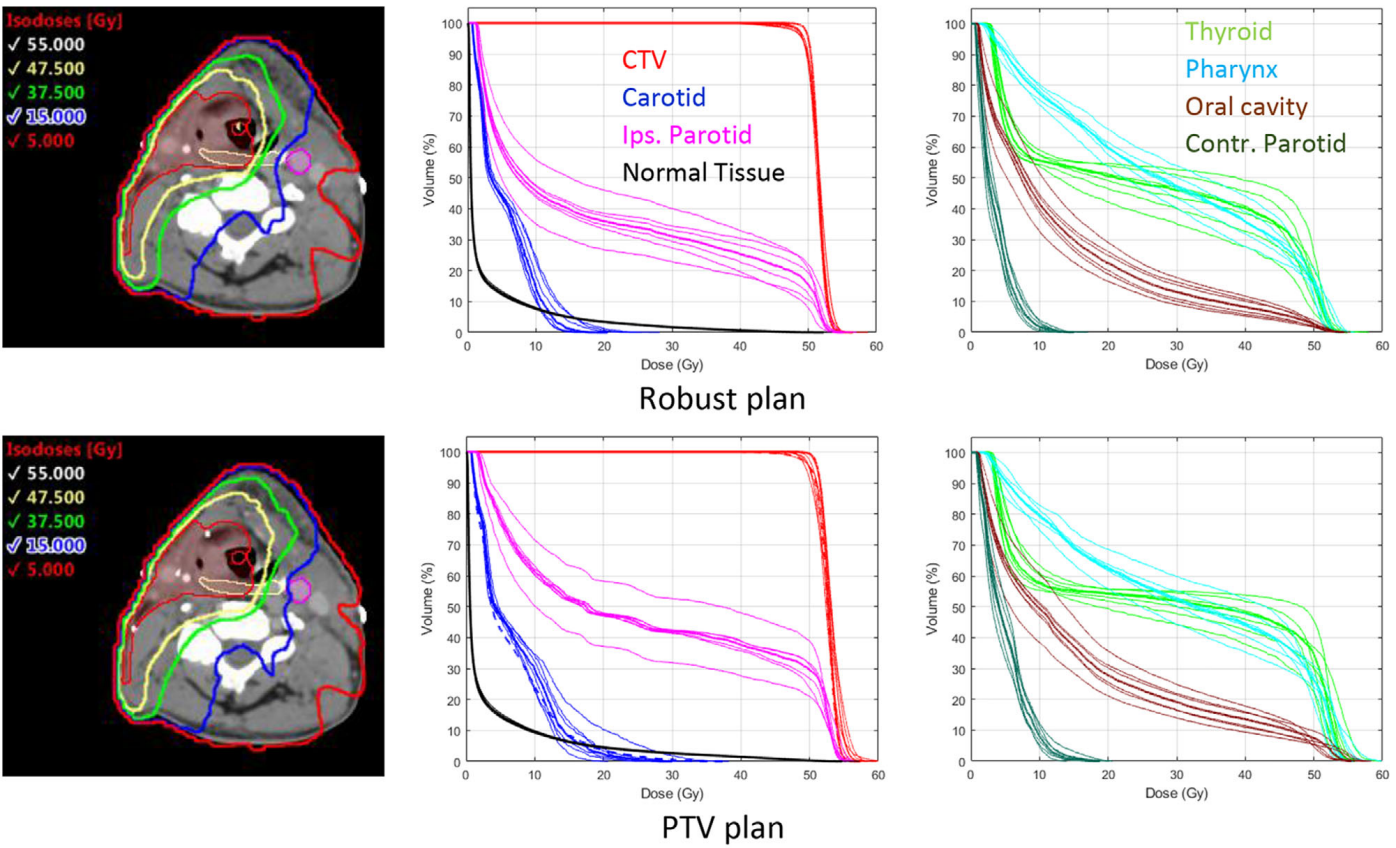
Left: Isodose distributions on nominal scenario for robust‐optimized (top row) and PTV‐based (bottom row) mixed‐beam radiotherapy (MBRT) plans for the first head and neck patient. Clinical target volume (CTV) is shown in red, contralateral carotid artery in pink and pharyngeal constrictors in yellow. Center/Right: Dose–volume histograms (DVHs) for nominal scenario (thick lines) and six setup error scenarios (thin lines). DVHs, for nominal scenario, for any PTV or planning at risk volume (PRV) structures are shown as thick dashed lines

Figure 5 shows a comparison of isodose distributions and dose–volume histograms (DVHs) for the robust‐optimized and PTV‐based MBRT plans for the first head and neck case. One hundred fifty apertures were used for both plans. The 37.5 Gy and 47.5 Gy (75% and 95% of the prescription dose, respectively) isodoses are more conformal to the CTV in the robust plan than in the PTV plan. Furthermore, the CTV DVHs for all scenarios are more closely spaced for the robust plan indicating that the CTV coverage is less sensitive to setup errors than the PTV plan.

The CTV coverage for all six error scenarios was clinically acceptable for both plans, however, for the PTV‐based plan, in five of the six error scenarios the *D*_5%_ of the CTV exceeded the clinical tolerance. Compared to the CTV, the OAR DVHs show a greater variation with setup errors. For the pharyngeal constrictors and the thyroid, the largest variations occurred for shifts along the left/right direction, while for the oral cavity and ipsilateral parotid gland the largest variation occurred with shifts along the superior/inferior direction. For the PTV‐based plan, the DVHs for the contralateral carotid artery show large deviations from the DVH for the corresponding PRV volume (blue dashed line) on the nominal case. The clinical tolerances for the carotid artery were exceeded in three out of the six error scenarios for the PTV‐based plan.

The dose–volume metrics for the nominal and six setup errors scenarios, for both the robust‐optimized and PTV‐based plans are summarized in Table 2. The robust‐optimized plan has a higher average CTV dose homogeneity index (HI_95/107_), 94.1% versus 67.5%, and its standard deviation is smaller compared to the PTV‐based plan. All of the OAR dose metrics are lower, by 20.4% on average, for the robust‐optimized plan than for the PTV‐based plan. The normal tissue *V*_10%_ was 4% lower for the robust plan. The number of MUs as well as the electron contributions in terms of MUs and mean CTV dose are similar for both plans.

**TABLE 2:** Comparison of dose–volume metrics (average ± SD) for nominal and six setup error scenarios for the two head and neck clinical cases. The larynx structure was not contoured on Patient 1

	**H&N Patient 1**		**H&N Patient 2**	
	**Robust**	**PTV**	**Robust**	**PTV**
CTV HI_95/107_ (%)	94.1 ± 3.1	67.5 ± 8.4	90.7 ± 2.1	80.0 ± 13.2
Contralateral carotid artery, *D*_mean_ (Gy)	5.4 ± 0.6	6.9 ± 0.9	5.9 ± 0.6	6.8 ± 0.9
Contralateral carotid artery, *D*_2%_ (Gy)	15.0 ± 2.5	20.9 ± 4.5	20.4 ± 5.5	25.6 ± 6.4
Ipsilateral parotid gland, *D*_mean_ (Gy)	19.3 ± 2.8	26.0 ± 2.9	19.0 ± 3.8	27.5 ± 4.0
Contralateral parotid gland, *D*_mean_ (Gy)	3.5 ± 0.4	4.9 ± 0.5	7.0 ± 0.4	7.7 ± 0.4
Glottic/supraglottic larynx, *D*_2%_ (Gy)	–	–	35.5 ± 5.5	47.7 ± 4.7
Thyroid, *D*_mean_ (Gy)	25.3 ± 2.5	28.7 ± 2.0	20.4 ± 2.3	25.1 ± 2.0
Pharyngeal constrictors, *D*_mean_ (Gy)	28.4 ± 1.5	31.0 ± 1.6	30.7 ± 2.0	36.1 ± 2.2
Oral cavity, *D*_mean_ (Gy)	13.1 ± 1.9	15.9 ± 2.0	16.3 ± 1.2	20.1 ± 1.6
Normal tissue, *V*_10%_	14.7 ± 0.6	18.7 ± 0.7	14.3 ± 0.4	17.6 ± 0.5
Total MU	744.6	763.5	876.7	842.1
Electron MU fraction (%)	22.9	22.5	19.5	22.6
Electron CTV *D*_mean_ fraction (%)	35.6	35.9	21.5	34.1

*Note*: Bold values indicate the "best" value between the two plans for each patient.

Abbreviation: CTV, clinical target volume; MU, monitor unit.

**FIGURE 6:**
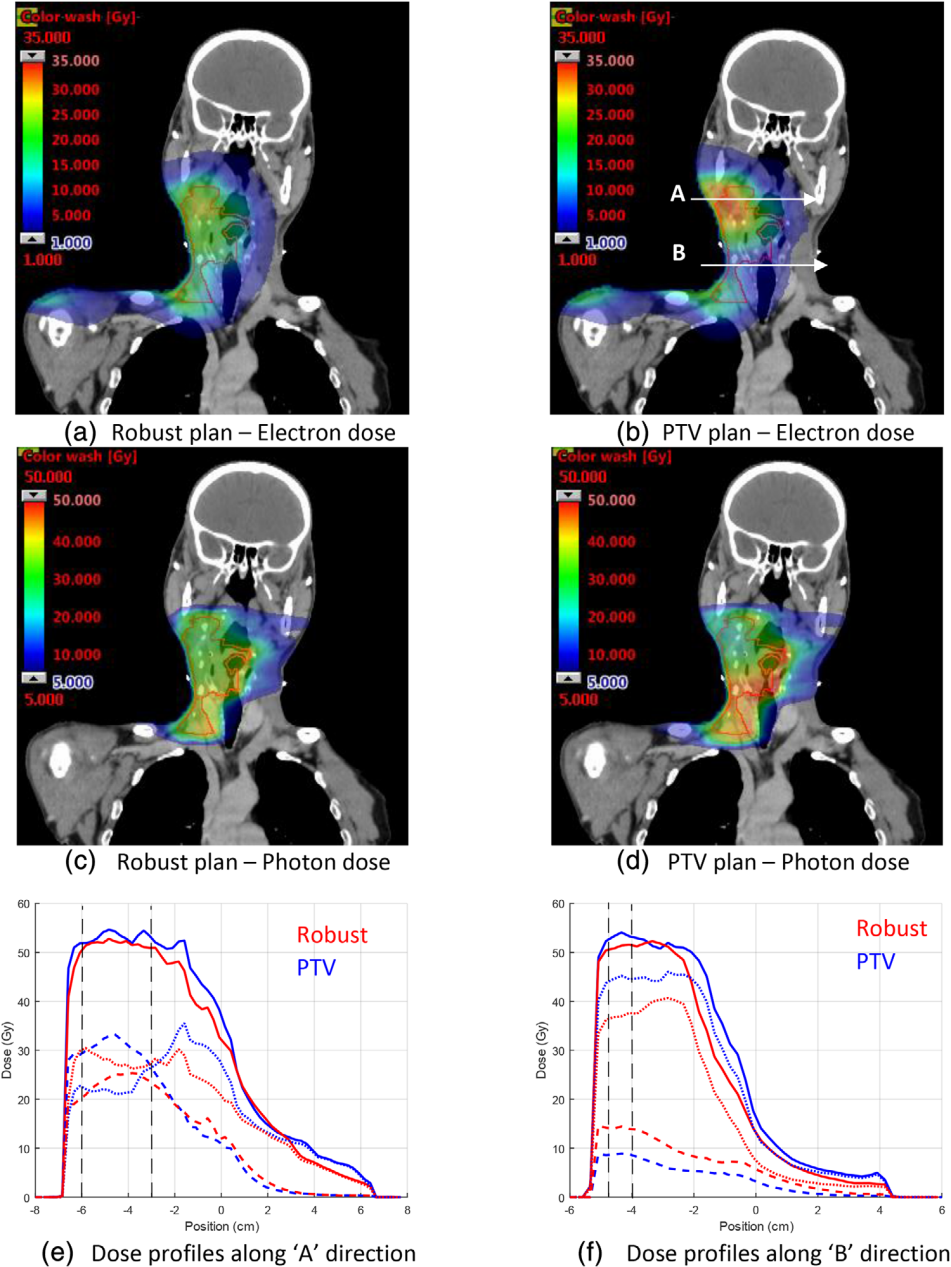
Comparison of electron (a,b) and photon (c,d) dose contribution for robust‐optimized (left column) and PTV‐based (right column) mixed‐beam radiotherapy (MBRT) plans. Electron, photon, and total dose profiles (e,f) for the directions indicated by the white arrows in (b). Solid lines indicate the total dose, thick dashed lines are electron dose, and fine dashed lines are photon dose. Vertical dashed lines indicate the extent of the clinical target volume (CTV)

Figure 6 shows the dose distributions from the electron apertures or photon apertures only for the two plans. The dose distributions for both modalities are more uniform for the robust plan than for the PTV plan. Dose profiles along the left/right direction are included for two different positions, corresponding to the maximum and minimum electron dose contribution for the PTV‐based plan. The relative electron dose contributions at these different locations vary, but the range of the electron dose profile in Figure 6e appears to be greater for the robust plan than for the PTV‐based plan. This observation is consistent with the finding that the MU‐weighted mean energy of the robust plan is 16.0 MeV and the beam energy with the most MUs is 22 MeV, while the corresponding values for the PTV‐based plan are 15.5 MeV and 12 MeV. Comparing the photon dose profiles in Figure 6f, it appears that the tighter distal margin for the robust plan is determined mainly by the photon contribution of this plan.

#### 3.2.2 Head and neck case 2

**FIGURE 7:**
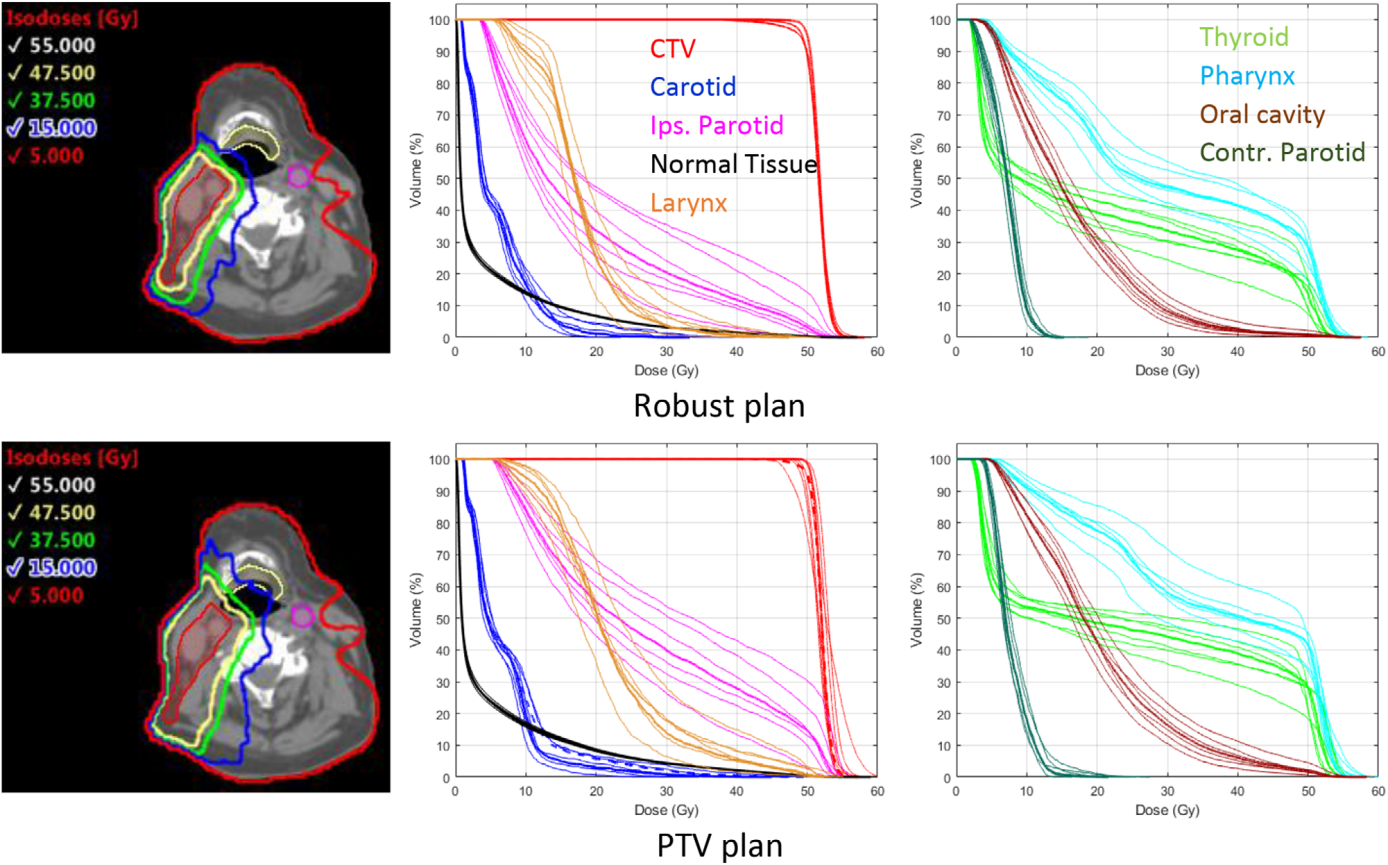
Left: Isodose distributions on nominal scenario for robust‐optimized (top row) and PTV‐based (bottom row) mixed‐beam radiotherapy (MBRT) plans for the second head and neck patient. Clinical target volume (CTV) is shown in red, contralateral carotid artery in pink and larynx in yellow. Center/Right: Dose–volume histograms (DVHs) for nominal scenario (thick lines) and six setup error scenarios (thin lines). DVHs, for nominal scenario, for any PTV or planning at risk volume (PRV) structures are shown as thick dashed lines

Figure 7 shows the isodose distributions and DVHs for second head and neck case which also used 150 apertures for both plans. Once again, the isodoses are more conformal to the CTV for the robust plan than for the PTV‐based plan. For the PTV‐based plan, the CTV DVHs vary noticeable across the six error scenarios and deviate from the nominal PTV DVH (red dashed line). The largest deviation corresponds to a 5 mm shift in the superior direction. For this case, the CTV *D*_95%_ was below the clinical tolerance. The CTV *D*_5%_ as well as some of the carotid plan criteria were exceeded in two of the tested error scenarios. The dose–volume metrics in Table 2 show that similar to the first head and neck patient case, the average CTV dose homogeneity was lower and had a larger standard deviation for the PTV‐based plan (80.0 ± 13.2%) compared to the robust‐optimized plan (90.7 ± 2.1%). Table 2 also shows that the OAR doses were consistently lower, by on average 19%, for the robust‐optimized plan and the normal tissue *V*_10%_ was 3.3% lower. The fraction of the CTV mean dose contributed by the electron apertures was lower (21.5% vs. 34.1%) for the robust plan compared to the PTV‐based plan.

**FIGURE 8:**
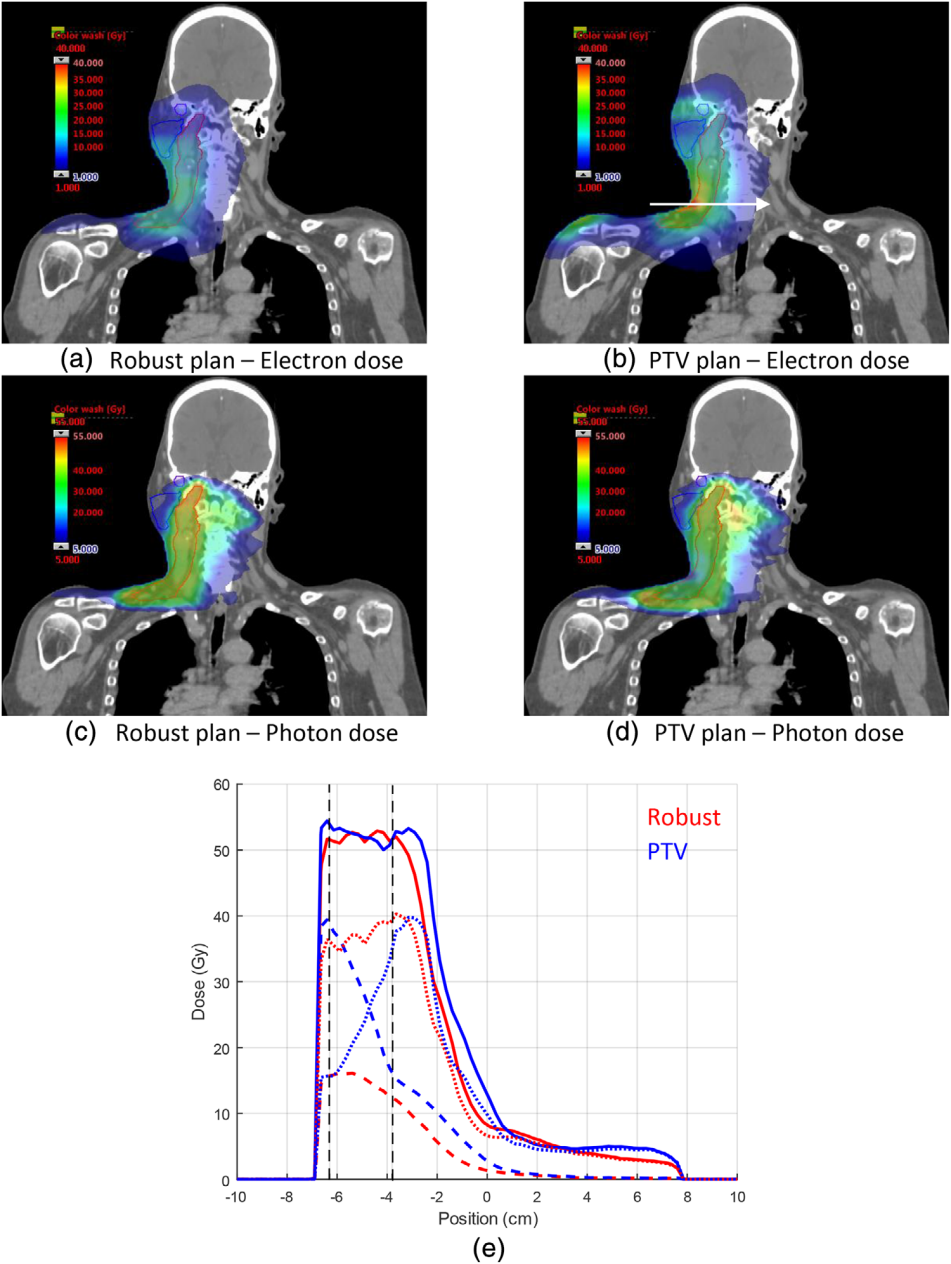
Comparison of electron (a,b) and photon (c,d) dose contributions for second head and neck case; (e) electron (thick dashed line), photon (thin dashed line) and total (solid lines) dose profiles for the direction indicated by the white arrow in (b). Vertical dashed lines indicate the extent of the clinical target volume (CTV)

Figure 8 shows the photon and electron contributions for both plans. Similar to the first head and neck case, the electron dose distribution is more uniform for the robust plan compared to the PTV‐based plan. A lateral dose profile at the location of the maximum electron dose contribution in the PTV‐based plan shows a reduced electron dose for the robust‐plan as well as a tighter distal margin for the photon dose profile. Similar trends were noted for dose profiles at other locations. The mean MU‐weighted energy of the robust plan is 17.8 MeV and the beam energy with most MUs is 22 MeV, while the corresponding values for the PTV‐based plan are 15.6 MeV and 15 MeV.

#### 3.2.3 Brain case

**FIGURE 9:**
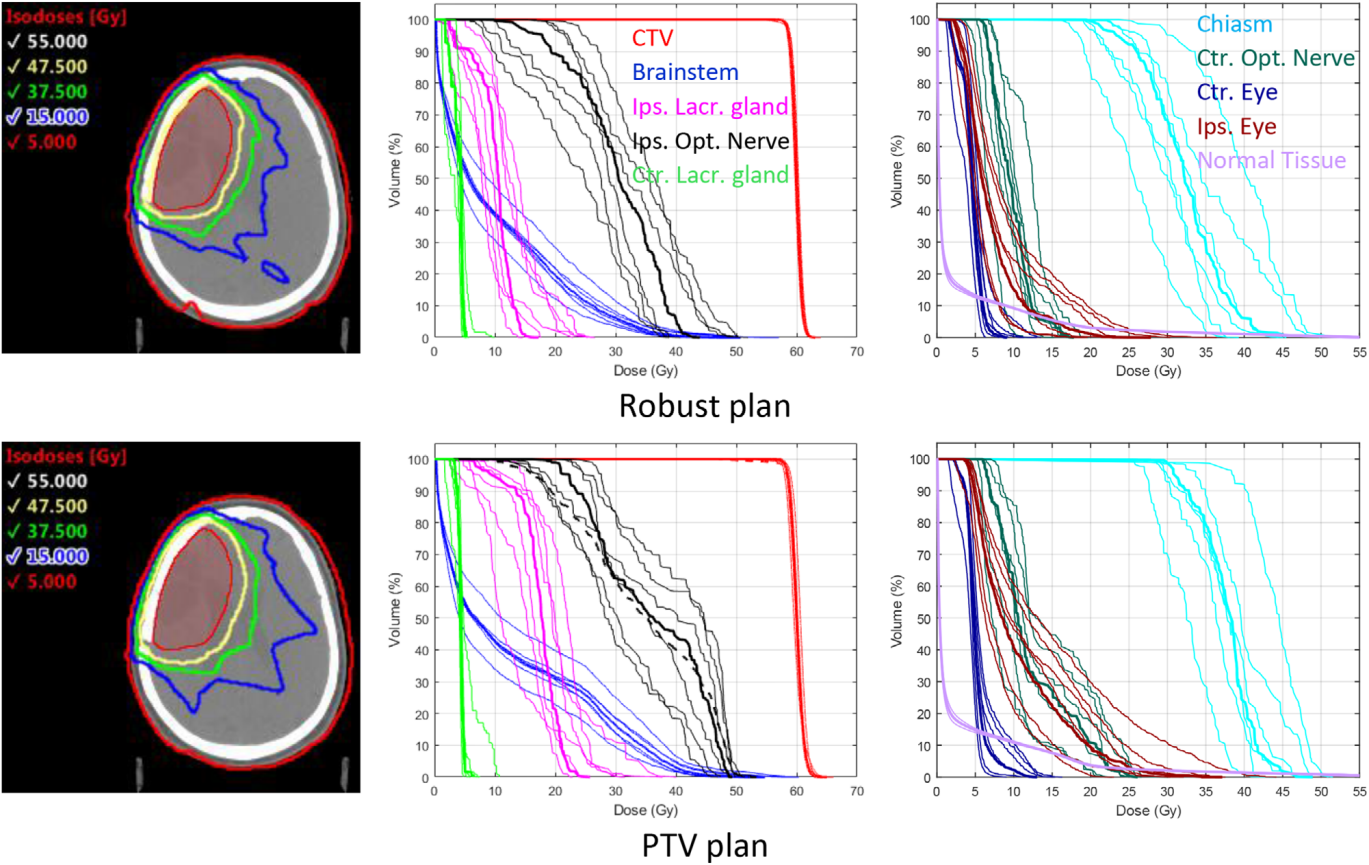
Left: Isodose distributions on nominal scenario for robust‐optimized (top row) and PTV‐based (bottom row) mixed‐beam radiotherapy (MBRT) plans for the brain patient case. The clinical target volume (CTV) is shown in red. Center/Right: Dose–volume histograms (DVHs) for nominal scenario (thick lines) and six setup error scenarios (thin lines). DVHs, for nominal scenario, for any PTV or planning at risk volume (PRV) structures are shown as thick dashed lines

Figure 9 shows the isodose distributions and DVHs for the brain case. Both the robust and PTV plans used 80 apertures. Table 3 summarizes the relevant dose–volume metrics for these plans. The CTV dose homogeneity index was almost identical for both plans, while the OAR doses were 21.7% lower, on average, for the robust plan. The normal tissue *V*_10%_ was 4% lower for the robust plan compared to the PTV plan. Clinical dose–volume criteria were respected for all setup error scenarios for both plans.

**TABLE 3:** Comparison of dosimetric values (average ± SD) across nominal and six setup error scenarios for the brain case

	**Robust**	**PTV**
Number of apertures	60	60
CTV HI_95/107_ (%)	99.7 ± 0.3	99.6 ±0.3
Chiasm, *D*_2%_ (Gy)	41.6 ± 4.9	44.7 ± 3.8
Brainstem, *D*_2%_ (Gy)	36.4 ± 2.8	42.6 ± 3.6
Ipsilateral optic nerve, *D*_2%_ (Gy)	42.3 ± 5.4	47.9 ± 2.0
Contralateral optic nerve, *D*_2%_ (Gy)	15.0 ± 2.8	23.1 ± 2.3
Ipsilateral lacrimal gland, *D*_mean_ (Gy)	10.7 ± 2.3	17.6 ± 3.2
Contralateral lacrimal gland, *D*_mean_ (Gy)	3.9 ± 0.5	4.4 ± 0.6
Ipsilateral eye, *D*_2%_ (Gy)	18.3 ± 5.9	27.2 ± 6.1
Contralateral eye, *D*_2%_ (Gy)	7.9 ± 1.9	10.1 ± 2.7
Normal tissue, *V*_10%_ (%)	14.9 ± 0.4	18.9 ± 0.5
Total MU	431.7	434.3
Electron MU fraction (%)	16.2	7.2
Electron CTV *D*_mean_ fraction (%)	31.6	12.8

*Note*: Bolded values indicate the “best” value between the two plans.

Abbreviation: CTV, clinical target volume; MU, monitor unit.

**FIGURE 10:**
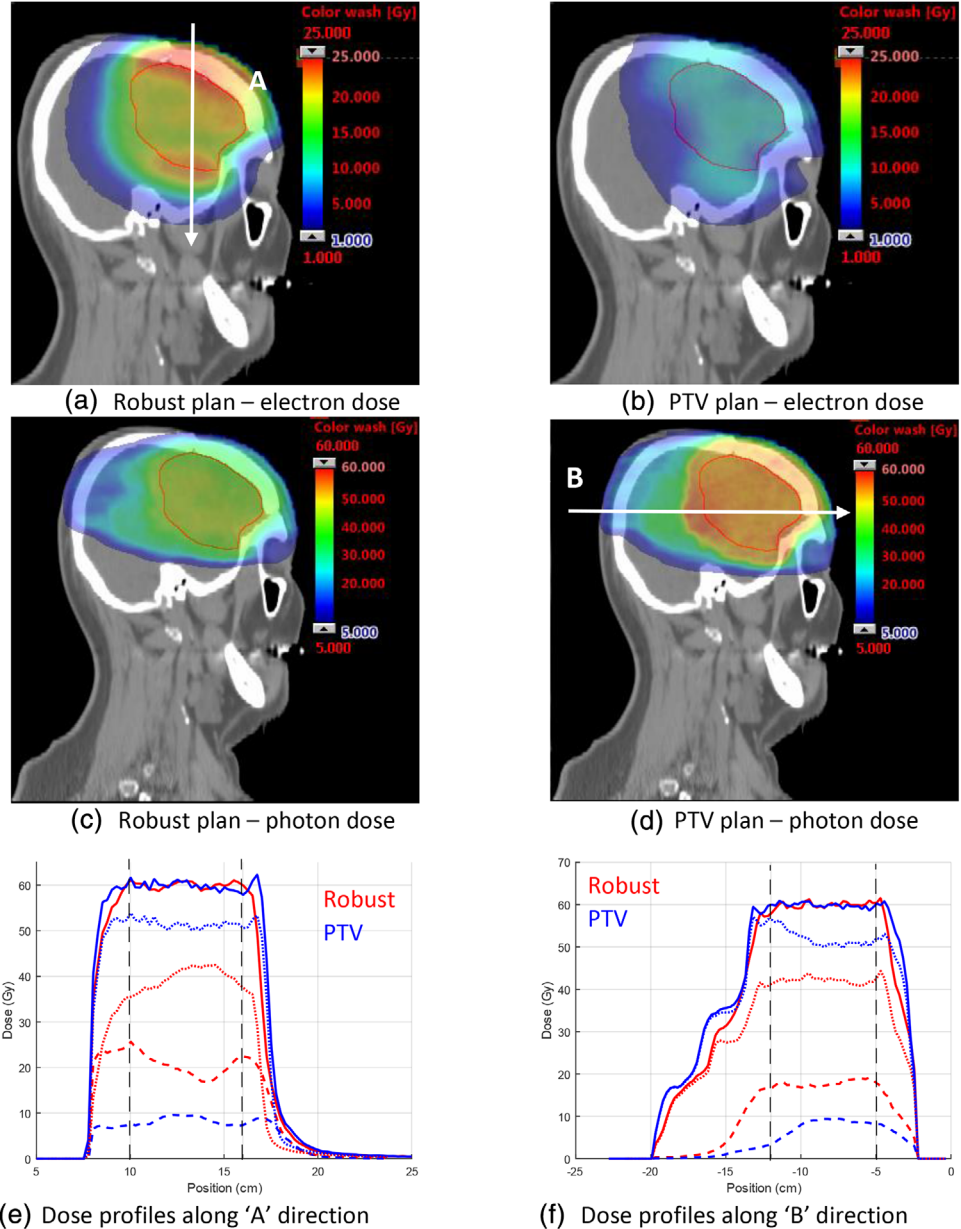
Comparison of electron (a,b) and photon (c,d) dose contributions for the brain case. Electron, photon, and total dose profiles (e,f) for the direction indicated by the white arrows in (a) and (d). Solid lines indicate the total dose, thick dashed lines are electron dose, and fine dashed lines are photon dose. Vertical dashed lines indicate the extent of the clinical target volume (CTV)

The electron dose contribution for the robust plan is approximately 2.5 times greater than for the PTV plan. Figure 10 compares the electron and photon dose contribution of the two plans. For this case, the electron dose contribution for the robust plan is less uniform than for the PTV‐based plan. The robust plan has an increased electron dose contribution at the superior and inferior borders of the CTV. The enhancement on the superior border is inside the skull. This is evident in the electron dose profile for the robust plan (red dashed line) in Figure 10e. Another observation from Figure 10e,f is that the photon dose contribution of the robust plan contributes to the smaller margin on the inferior side of the CTV. The mean MU‐weighted energy of the PTV‐based plan for the brain case was 20.6 MeV and the beam energy with the most MUs was 22 MeV, while the robust plan used only 22 MeV beams.

## 4 DISCUSSION

In this paper, we presented the development, experimental validation, and application of a robust hybrid DAO approach for MBRT planning. Similar to Renaud et al.,^12^ we investigated robustness to translational setup errors using the stochastic optimization approach. However, our approach uses a combination of simulated annealing and column generation, while Renaud's approach was based on column generation only.

Compared to the previous experimental validation of the delivery of (nonrobust) MBRT plans by Mueller et al.,^6^ the agreement between film measurements and dose calculations for our robust plans was slightly lower, at 97.7% for 3%/2 mm criterion, but still acceptable according to the recommendations of AAPM Task Group 218.^25^ The photon component of the MBRT plans previously validated by Mueller et al. consisted of non‐coplanar arcs, while in the current study we used a coplanar step‐and‐shoot delivery for photons and electrons. We also presented validation results for photon and electron apertures separately, while previous work compared only the total dose distribution. The agreement between calculations and measurements was found to be lower for the electron apertures than for the photon apertures. This could be attributed to limitations of the electron beam model used in dose calculations as well as a higher sensitivity of electron beams to experimental uncertainties such as air gaps.^27^ The agreement between calculations and measurements was similar for the shifted and unshifted cases which gives us confidence that setup errors are correctly modeled. Overall, these experiments demonstrate the feasibility and accuracy of the delivery of MBRT plans using the photon MLC and a shortened SSD. However, before clinical implementation, a more comprehensive evaluation of the delivery accuracy of robust MBRT plans is needed. Heng et al.^28^ recently reported initial results of a patient‐specific MBRT QA process based on ion chamber and film dosimetry. Our group has also developed a patient‐specific QA procedure for MBRT using log file based dose calculation and EPID measurements.

A comparison of robust‐optimized and PTV‐based plans was carried out for two head and neck and one brain clinical case. Similar to Renaud et al.,^12^ we found that PTV‐based MBRT plans were not robust to translational setup errors and that robust‐optimized plan had improved OAR sparing. We also found a trend of increased electron beam energies in the robust plans. However, while Renaud et al. noted an increase in the electron dose contribution for the robust plans compared to the PTV‐based plans we did not find a consistent trend in our plans. The electron contributions were similar for both plans for the head and neck cases, while for the brain case the electron contribution in the robust plan was more than double that for the PTV‐based plan. Also, the dose distribution from the electron apertures of the robust plan was less uniform for the brain case compared to the head and neck cases. The enhancement at the superior side of the CTV coincides with the skull, where the increased stopping power in bone is expected to result in a dose enhancement. However, the dose enhancement at the inferior side of the CTV does not coincide with bone. Therefore, we hypothesize that in the robust plan the optimizer is increasing the fluence at the edges of the target volume to compensate for the setup errors. This is consistent with the edge enhancement noted by Unkelbach and Oelfke^29^ when determining the ideal fluence profiles accounting for target volume displacements in an idealized geometry. Compared to the head and neck cases, there are fewer dose‐limiting OARs surrounding the target volume for the brain case which might explain why the edge enhancement was not observed for the head and neck cases. It should be noted that both Renaud's and our study used a limited number of patient cases and a larger number of cases is needed to clearly identify trends and the factors that influence them.

## 5 CONCLUSIONS

A framework for robust optimization of deliverable MBRT plans has been developed and validated. Plans optimized with our robust framework were compared with PTV‐based plans on two head and neck and one brain case in terms of robustness of target volume dose to setup errors as well as OAR sparing. PTV‐based MBRT were found to not be robust to setup errors, while the dose delivered by the robust‐optimized plans were clinically acceptable for all error scenarios and had better OAR sparing. This study shows that the robust optimization is a promising alternative to conventional PTV margins for MBRT.

## CONFLICT OF INTEREST

The authors declare no conflict of interest.

## Data Availability

The data that support the findings of this study are available on request from the corresponding author. The data are not publicly available due to privacy or ethical restrictions.

## References

[1] Al‐Yahya K , Schwartz M , Shenouda G , Verhaegen F , Freeman C , Seuntjens J . Energy modulated electron therapy using a few leaf electron collimator in combination with IMRT and 3D‐CRT: Monte Carlo‐based planning and dosimetric evaluation. Med Phys. 2005;32(9):2976‐2986. 10.1118/1.2011089. 16266112

[2] Alexander A , Soisson E , Renaud MA , Seuntjens J . Direct aperture optimization for FLEC‐based MERT and its application in mixed beam radiotherapy. Med Phys. 2012;39(8):4820‐4831. 10.1118/1.4736423 22894408

[3] Korevaar EW , Huizenga H , Stroom JC , Willem Leer JH , Brahme A . Investigation of the added value of high‐energy electrons in intensity‐modulated radiotherapy: four clinical cases. Int J Radiat Oncol Biol Phys. 2002;52(1):236‐253. 11777643 10.1016/s0360-3016(01)02689-x

[4] Palma BA , Sánchez AU , Salguero FJ , et al. Combined modulated electron and photon beams planned by a Monte‐Carlo‐based optimization procedure for accelerated partial breast irradiation. Phys Med Biol. 2012;57(5):1191‐1202. 10.1088/0031-9155/57/5/1191 22330241

[5] Xiong W , Li J , Chen L , et al. Optimization of combined electron and photon beams for breast cancer. Phys Med Biol. 2004;49(10):1973‐1989. 10.1088/0031-9155/49/10/010 15214536

[6] Mueller S , Manser P , Volken W , et al. Part 2: Dynamic mixed beam radiotherapy (DYMBER): photon dynamic trajectories combined with modulated electron beams. Med Phys. 2018;45(9):13085. 10.1002/mp.13085 29992574

[7] Mueller S , Fix MK , Joosten A , et al. Simultaneous optimization of photons and electrons for mixed beam radiotherapy. Phys Med Biol. 2017;62(14):5840‐5860. 10.1088/1361-6560/aa70c5 28467321

[8] Renaud MA , Serban M , Seuntjens J . On mixed electron–photon radiation therapy optimization using the column generation approach. Med Phys. 2017;44(8):4287‐4298. 10.1002/mp.12338 28500783

[9] Al‐Yahya K , Hristov D , Verhaegen F , Seuntjens J . Monte Carlo based modulated electron beam treatment planning using a few‐leaf electron collimator—feasibility study. Phys Med Biol. 2005;50(5):847‐857. 10.1088/0031-9155/50/5/009 15798259

[10] Ma CM , Pawlicki T , Lee MC , et al. Energy‐ and intensity‐modulated electron beams for radiotherapy. Phys Med Biol. 2000;45(8):2293‐2311. 10.1088/0031-9155/45/8/316 10958195

[11] du Plessis FCP , Leal A , Stathakis S , Xiong W , Ma CM . Characterization of megavoltage electron beams delivered through a photon multi‐leaf collimator (pMLC). Phys Med Biol. 2006;51(8):2113‐2129. 10.1088/0031-9155/51/8/011 16585849

[12] Renaud MA , Serban M , Seuntjens J . Robust mixed electron–photon radiation therapy optimization. Med Phys. 2019;46(3):1384‐1396. 10.1002/mp.13381 30628079

[13] Fredriksson A . A characterization of robust radiation therapy treatment planning methods: from expected value to worst case optimization. Med Phys. 2012;39(8):5169‐5181. 10.1118/1.4737113 22894442

[14] Mueller S , Risse T , Fix MK , et al. EP‐1930: mixed beam radiotherapy for sternum and lung treatments. Radiother Oncol. 2018;127:S1049. 10.1016/s0167-8140(18)32239-4

[15] Fix MK , Manser P , Frei D , Volken W , Mini R , Born EJ . An efficient framework for photon Monte Carlo treatment planning. Phys Med Biol. 2007;52(19):N425‐N437. 10.1088/0031-9155/52/19/N01 17881793

[16] Kawrakow I , Fippel M . VMC++: a fast MC algorithm for radiation treatment planning. The Use of Computers in Radiation Therapy. Springer; 2000:126‐128. 10.1007/978-3-642-59758-9_46

[17] Henzen D , Manser P , Frei D , et al. Monte Carlo based beam model using a photon MLC for modulated electron radiotherapy. Med Phys. 2014;41(2):021714. 10.1118/1.4861711 24506605

[18] Fix MK , Frei D , Volken W , Neuenschwander H , Born EJ , Manser P . Monte Carlo dose calculation improvements for low energy electron beams using eMC. Phys Med Biol. 2010;55(16):4577‐4588. 10.1088/0031-9155/55/16/S11 20668339

[19] Neuenschwander H , Born EJ . A macro Monte Carlo method for electron beam dose calculations. Phys Med Biol. 1992;37(1). 10.1088/0031-9155/37/1/007

[20] Neuenschwander H , MacKie TR , Reckwerdt PJ . MMC‐a high‐performance Monte Carlo code for electron beam treatment planning. Phys Med Biol. 1995;40(4):543‐574. 10.1088/0031-9155/40/4/005 7610114

[21] Romeijn HE , Ahuja RK , Dempsey JF , Kumar A . A column generation approach to radiation therapy treatment planning using aperture modulation. SIAM J Optim. 2005;15(3):838‐862. 10.1137/040606612

[22] Terribilini D , Manser P , Frei D , Volken W , Mini R , Fix K . Implementation of a brachytherapy Ir‐source in an in‐house system and comparison of simulation results with EGSnrc, VMC++ and PIN. J Phys Conf Ser. 2007;74(1). 10.1088/1742-6596/74/1/021022

[23] Lewis D , Micke A , Yu X , Chan MF . An efficient protocol for radiochromic film dosimetry combining calibration and measurement in a single scan. Med Phys. 2012;39(10):6339‐6350. 10.1118/1.4754797 23039670 PMC9381144

[24] Lewis D , Chan MF . Correcting lateral response artifacts from flatbed scanners for radiochromic film dosimetry. Med Phys. 2015;42(1):416‐429. 10.1118/1.4903758 25563282 PMC5148133

[25] Miften M , Olch A , Mihailidis D , et al. Tolerance limits and methodologies for IMRT measurement‐based verification QA: recommendations of AAPM Task Group No. 218. Med Phys. 2018;45(4):e53‐e83. 10.1002/mp.12810 29443390

[26] Thilmann C , Sroka‐Perez G , Krempien R , Hoess A , Wannenmacher M , Debus J . Inversely planned intensity modulated radiotherapy of the breast including the internal mammary chain: a plan comparison study. Technol Cancer Res Treat. 2004;3(1):69‐75. 10.1177/153303460400300108 14750895

[27] Gerbi BJ , Antolak JA , Deibel FC , et al. Recommendations for clinical electron beam dosimetry: supplement to the recommendations of Task Group 25. Med Phys. 2009;36(7):3239‐3279. 10.1118/1.3125820 19673223

[28] Heng VJ , Serban M , Seuntjens J , Renaud M . Ion chamber and film‐based quality assurance of mixed electron‐photon radiation therapy. Med Phys. 2021;48(9):5382‐5395. 10.1002/mp.15081 34224144

[29] Unkelbach J , Oelfke U . Inclusion of organ movements in IMRT treatment planning via inverse planning based on probability distributions. Phys Med Biol. 2004;49(17):4005‐4029. 10.1088/0031-9155/49/17/013 15470920

